# “Guboow”—child abuse or traditional medical treatment? a case report

**DOI:** 10.1007/s00414-021-02513-2

**Published:** 2021-02-15

**Authors:** N. Lange-Herr, A. Rindlisbacher, F. Romano, C. Jackowski

**Affiliations:** 1 Institute of Forensic Medicine, University of Bern, Bühlstrasse 20, 3012 Bern, Switzerland; 2 Pediatric Emergency Department, University Children’s Hospital, Inselspital, University of Bern, Bern, Switzerland

**Keywords:** Traditional medical treatment, Child abuse, Case report, Burn injury, Guboow

## Abstract

The examination of children suspected of being abused poses a great challenge for forensic pathologists. The risk of misjudgment is high and can have serious consequences for the child and the family. In unclear cases, an assessment should always be carried out on an interdisciplinary basis with the involvement of the relevant disciplines such as pediatrics, dermatology, or radiology. We present the case of a 2.5-year-old boy who was presented by his parents at the Pediatric Emergency Department of a Swiss University Hospital due to fever and weight loss. During the physical examination, conspicuous findings on the abdomen were present, and the responsible emergency physicians informed the child protective services. A clinical forensic examination occurred on behalf of the child protection services. The abdomen of the child showed several symmetrical scars. The initial questioning of the parents did not provide clear information about the origin of the injuries. Further professional questioning of the family by the child protective services concluded that the injuries were the result of a traditional medical treatment in Somalia, which occurred several weeks before.

## Case circumstances

A family of Somali origin, which has been living in Switzerland for several years, presented the 2.5-year-old son at the Pediatric Emergency Department of a Swiss University Hospital a few days after their return from a stay of several weeks in Somalia. The reported symptoms were fever for several days, vomiting, and weight loss. The physical examination revealed conspicuous scars on the abdomen, which were partly roundish and partly linear. When asked, the parents stated that the boy had scratched himself and that the round scars were the result of scalding several months ago. The further examination revealed no other suspicious findings. The child’s patient record confirmed a scalding 1 year before, however only affecting the right shoulder. Due to suspicion of child abuse, the child protective services were involved, and an examination by a forensic pathologist was commissioned.

## Physical examination

**Fig. 1:**
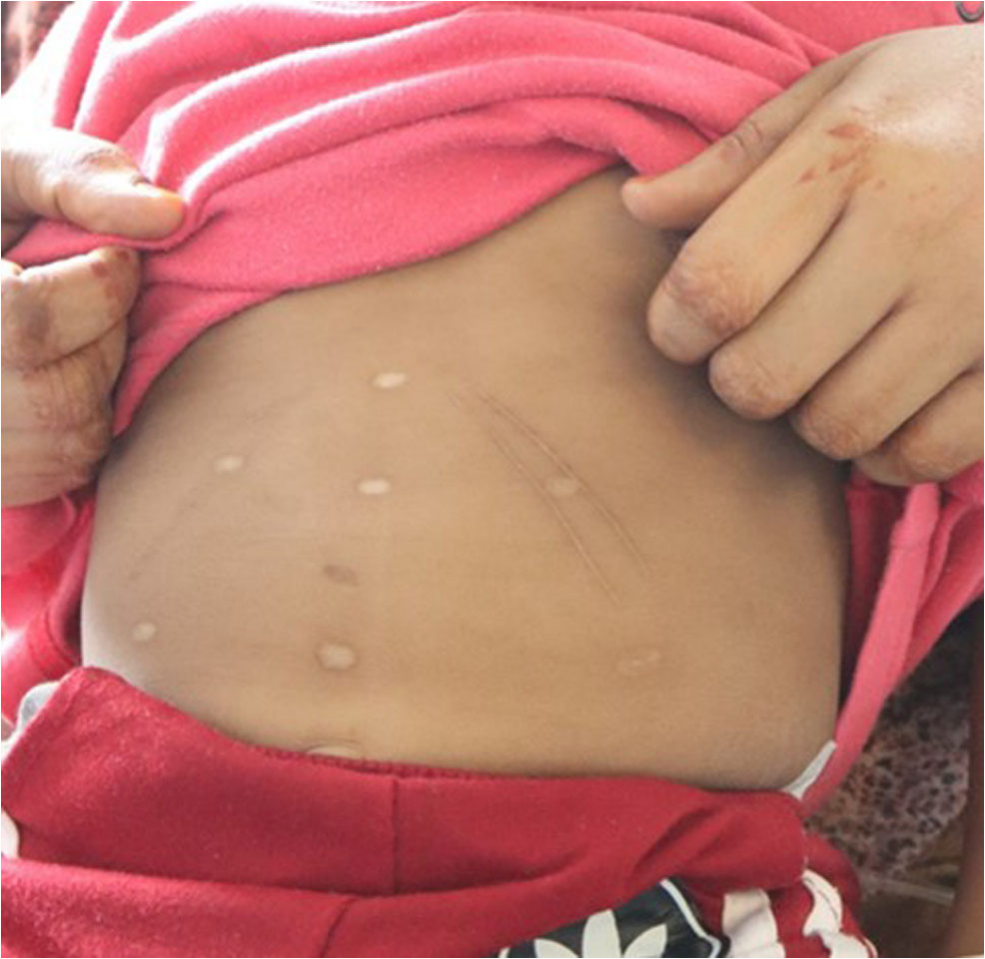
A 2.5-year-old boy with suspicious cicatricial lesions on the abdomen

**Fig. 2:**
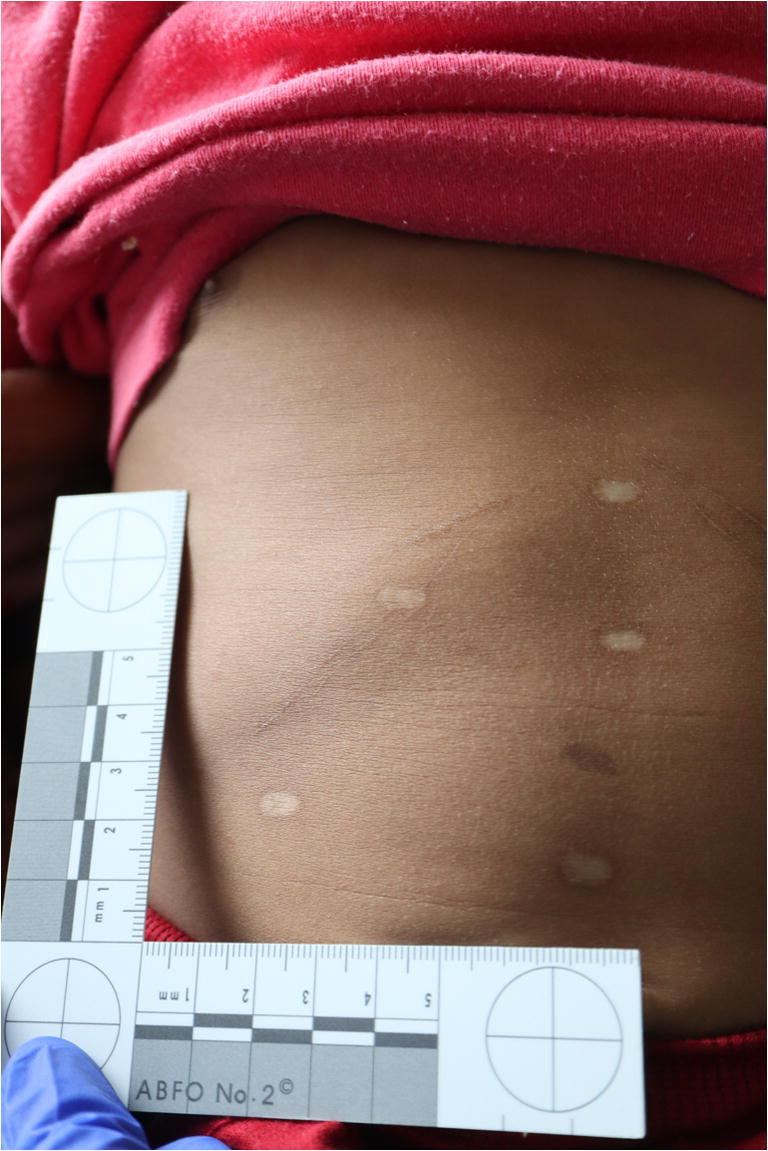
Symmetrical, round, and linear scars on the abdomen

**Fig. 3:**
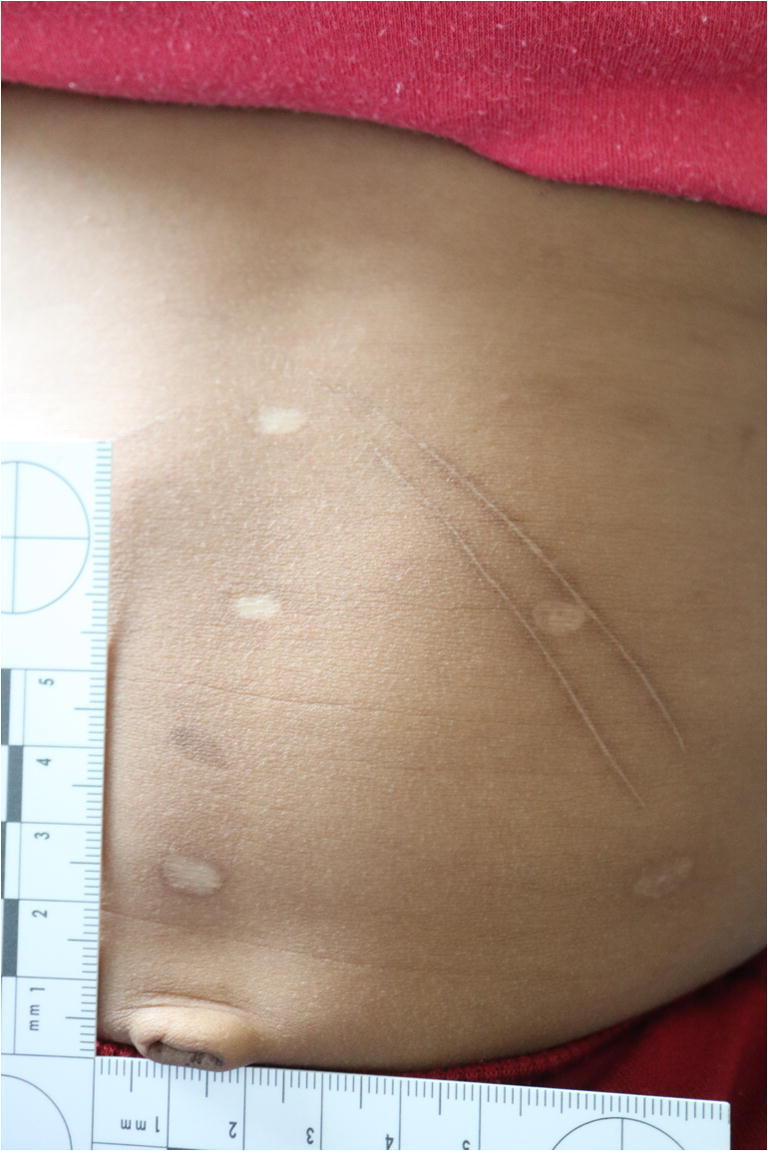
Symmetrical round and linear scars on the abdomen

The physical examination showed a boy weighing 12.3 kg (weight percentile: P10) in good nutritional condition. He presented agitated; his vital signs showed tachycardia and arterial hypertension. The abdominal findings are listed below (Figs. 1, 2, and 3):

- At least three linear, hyperpigmented, and centrally brightened scars on the right side of the abdomen, directly below the costal arch, following the course of the costal arch, up to a maximum of approximately 4-cm length.
- On the left side of the abdomen, directly below the costal arch, two linear hyperpigmented, centrally brightened scars, following the course of the costal arch, up to a maximum of approximately 7-cm length.
- On the abdomen, seven roundish-whitish scars, to a maximum of about 0.6 cm in diameter, symmetrically arranged in a triangle: Three scars were arranged approximately 3 cm above each other in the median line of the body, further two scars about 5 cm to the right and left of the lowest central scar, and the remaining two about 4 cm to the right and left of the middle central scar.

The rest of the body including the right shoulder showed no relevant findings, except for some unspecific grazes in exposed areas.

## Further investigations

Professional inquiry by the child protective services revealed that the mother had been in Somalia with the boy a few months ago. At that time, the boy had a swollen, painful abdomen. Due to COVID-19 travel restrictions, she was not able to return to Switzerland, and so she presented her son to a local traditional healer. She was asked to wait outside the examination room during the examination. When she heard her son scream, she entered the examination room and noticed that the healer had burned the boy’s abdomen. The mother stated that treating swelling and other diseases with localized burns is widespread in Somalia. However, she was not aware that such treatments are used on children. In an interview with the child protective services, the parents clearly distanced themselves from the traditional healer and assured that they would never again expose their child to such treatment. In consideration of the parents’ lack of intent to harm, the clinical absence of further signs of abuse, and the low risk of future exposure to traditional medicine, the child protective services refrained from a report to the public prosecutor’s office.

During a 4-day hospitalization, the initial tachycardia and arterial hypertension dissipated, and it was assumed that they were vegetative at the time of admission due to the child’s enormous fear and agitation towards the health personnel.

## Discussion

The assessment of cicatricial lesions is often challenging because the mechanism underlying the injury is often not clearly evident. Particularly in the case of child abuse, where the patient is often only able to provide limited information, a comprehensive assessment of the context and possible involvement of specialists from other disciplines is of great importance.

In the presented case, the morphology of the roundish scars suggested that they were the result of burns, and the symmetry of the injuries in an almost pyramid-like arrangement made an accidental burn unlikely. In this regard, the first working diagnosis at the Pediatric Emergency Department was the result of a ritual on the child’s abdomen in the context of child abuse.

Only through a professional and accurate interview with the family by the child protective services, it could be concluded that the scars were the result of Somalian traditional medical treatment.

Such treatments are widespread in East African countries (Somalia, Ethiopia, Sudan, Eritrea) [1] and are also known as “Guboow” [2–4]. “Guboow" means in Somali “scar” [2], “burning” [5], or also refers directly to the scar as a result of the treatment [3]. This practice is most common in rural areas and among Sunni people [2, 3].

The belief underlying the treatments is that the influences causing the disease must be destroyed by fire [2, 3]. Fire and disease cannot be in one body at the same time [1]. “Guboow” burns are usually caused by burning wood, and the burned area is directly related to the sick body part [2, 4]. The principle of using severe acute pain to treat chronic pain is also mentioned [6].

Similar injuries have been reported in Asian countries (Cambodia, Laos, Pakistan), where metal wires, coins, yarn, and other materials are also commonly used [4, 6, 7].

Possible hints for such a cause of a conspicuous skin alteration may be the location of the injury (defined area, symmetrical arrangement), approximately the same age of the findings and the information’s given by the patient or relatives [2, 3].

In some cases, such therapeutic burns can also lead to serious injuries such as sepsis, extensive skin defects or keloid, and death as well [6].

Overall, our literature search revealed only sparse reports of such treatments in the forensic and pediatric literature and isolated reports from the field of dermatology. However, widespread use of these practices in the treatment of children was apparent [2–4, 7].

To our knowledge, there are no precedents for the assessment of sequelae of traditional healing methods as child abuse. Several reports around the world describe initially suspicious skin changes that were later found to be the result of traditional healing methods without being classified as child abuse [7–12]. In the literature, consequences of traditional healing methods are usually defined as a differential diagnosis of child abuse and not as a subset of the latter [11, 12]. It should be noted that most of the treatment methods cited seem not to lead to permanent skin changes and have little potential for severe medical complications [8–11]. However, with regard to our patient, the lack of an informed consent of the mother to the Somali traditional healer performing the treatment and the multiple application of burns in a two-year-old conscious child can be considered child abuse, which, in addition to the scarring, led to conspicuous psychological consequences in our patient.

## Conclusion

Emergency physicians, pediatricians, dermatologists, and forensic pathologists should be aware of the existence of these practices. Especially in times of large demographic movements, it is the duty of medical professionals to consider the cultural and religious background of patients and, especially in the pediatric field, promote prevention through an explicit education of the families at risk. In the event of unclear examination results, the involvement of child protective services and forensic medicine should be considered.
